# A systematic review of mood and depression measures in people with severe cognitive and communication impairments following acquired brain injury

**DOI:** 10.1177/02692155221139023

**Published:** 2022-11-15

**Authors:** Alexandra E. Rose, Breda Cullen, Sarah Crawford, Jonathan J. Evans

**Affiliations:** 1 Institute of Health and Wellbeing, College of Medical, Veterinary and Life Sciences, University of Glasgow, Glasgow, UK; 2 Department of Psychology, 59386 Royal Hospital for Neuro-disability, London, UK

**Keywords:** Brain injury, mood assessment, depression, screening, severe brain injury

## Abstract

**Aim:**

A systematic review to identify which mood and depression measures are valid for use with people with severe cognitive and communication impairments following severe acquired brain injury.

**Method:**

A systematic search of Cochrane, Web of Science, Ovid, and EBSCOhost was performed in March 2020, July 2021, and September 2022. The search focused on self-report and observer-rated assessment tools used to assess mood, depression, and/or distress in those described as having a severe acquired brain injury. Psychometric properties were extracted using the Consensus-based standards for the selection of health measurement instruments (COSMIN) risk of bias checklist. Qualitative synthesis was performed on extracted patient data.

**Results:**

Nineteen papers detailing the psychometric properties of 25 measures were included, involving 2,914 participants. Nine papers provided details confirming the severity of participants’ cognitive and communication impairments. The remaining papers described including severely injured participants but provided limited details so that precise level of severity could not be confirmed. Only one paper showed evidence of adequate psychometric properties and included those with severe cognitive impairments in a study of two observer-rated measures, the Stroke Aphasia Depression Questionnaire (10 items) and the Aphasia Depression Rating Scale.

**Conclusions:**

Due to the exclusion of individuals with severe cognitive and communication consequences following brain injury, no studies using self-report measures showed adequate validity evidence to recommend their use in this population. A small study using two observer-rated scales included those with severe cognitive impairments and showed satisfactory evidence that these measures can be validly used with this population.

## Introduction

Distress, low mood, and depression are common following acquired brain injury.^1,2^ However, due to the overlap of symptoms of depression and the cognitive and physical consequences caused by brain injury (such as slowed processing, changes in sleep, appetite, or ability to engage in activities), it can be challenging to recognise mood difficulties after acquired brain injury.^3^ When the brain injury and its consequences are severe, the assessment of mood becomes more complex. This review focuses on the assessment of mood in people with severe and persistent impairments in cognition and communication following a brain injury.^4–6^

Low mood is traditionally assessed using self-report measures^7–10^ which require cognitive abilities such as reflecting on the past, comparing mood states, and comprehending complex and often abstract concepts such as mood, time, intensity, and “normal” versus “abnormal”. This makes them difficult to apply when cognitive and communication impairments are present. Other methods, such as observational measures, can also be challenging to use as mood-related behaviours may be clouded by the neurological symptoms of the brain injury, making it difficult to identify whether a mood issue is present.

To increase the likelihood of patients being offered proportional and appropriate treatment, it would be useful to clarify which mood assessment measures are valid for use with those with ongoing severe cognitive and communication impairments following a brain injury. Therefore, a systematic review of the evidence was undertaken. To the authors’ knowledge, no previous systematic review of mood measures for the population of people with severe brain injury has been completed.

## Objectives

The objectives of the systematic review were to establish:

- Which studies examining the validity of tools for the assessment of mood, distress, and depression in acquired brain injury include people with severe brain injury with persistent severe cognitive and communication impairments in their studies?
- How was severity defined by authors of the studies and what details regarding the degree of impairment of cognition and communication were provided?
- Which, if any, tools have been found to be reliable and valid measures of mood, distress, and depression when used with brain injured individuals with severe cognitive and communication impairments and what is the quality of the evidence for their reliability and validity?

## Method

A systematic review of the literature based upon a pre-registered protocol (PROSPERO identifier: CRD42019162649) was conducted according to PRISMA standards.^11^ The University of Glasgow maintained responsibility for the integrity and conduct of this review. This study was part of a PhD project funded by the Francis and Augustus Newman foundation.

Scoping studies were performed in December 2019 and January 2020 to ascertain the sensitivity of the terms and associated abbreviations for “depression”, “mood”, “brain injury”, and “assessment”. Initial scoping searches of validation studies showed that the term “severe brain injury” was not fully or consistently defined in published studies. It was also noted that although authors of papers described the included population as “severe”, patients with severe comprehension impairments were often excluded from validation studies and the exclusion was not always explicitly stated. Limiting search terms to those papers including the term “severe” was too restrictive and did not retrieve relevant papers. Additionally, limiting searches to titles excluded key papers in the literature. Thus, a broad search was applied in order to capture the appropriate literature and avoid missing relevant papers. Search terms were refined and searches were completed in March 2020 and repeated in July 2021 and September 2022 (see Appendix II for detailed search terms).

The following databases were searched from inception:

- Cochrane Library: Cochrane Database for Systematic Reviews (CDSR) and Cochrane Central Register of Controlled Trials (CENTRAL)
- Web of Science: Core collection and MEDLINE databases
- Ovid: Health and Psychosocial Instruments and Embase databases
- EBSCOhost: CINAHL, PsycARTICLES, PsycINFO and Psychology and Behavioural Sciences databases

This was supplemented by hand searching the references in the identified papers and any review papers on similar topics. The emphasis of the review was on published, peer-reviewed journals. Grey literature beyond articles derived from hand searched articles was not included. There were no restrictions in terms of the language of the published studies.

### Initial inclusion/exclusion criteria

Quantitative studies including standardisation studies, validation studies, diagnostic studies, cohort studies, case control studies, experiments and randomised control trials were included, if a method for mood assessment or measurement was used within the study. Papers that were not investigating the validity of the mood measure, or where data regarding the validity of the measure could not be extracted, were excluded.

Adult population studies (aged 18 or over) with acquired brain injury (inclusive of stroke, traumatic brain injury, hypoxic injury, and other forms of non-progressive brain injury) were included. Paediatric populations and adolescent populations (under the age of 18) and progressive neurological conditions (e.g., Parkinson's disease, multiple sclerosis, and dementia) were excluded.

Studies were selected if (1) the study population was defined by the study authors as adults with severe brain injury, or (2) the severity of injury was listed as a Glasgow Coma Scale (GCS) score of 8 or less, and/or post traumatic amnesia (PTA) period of more than 1 day, and/or (3) there was evidence of the presence of severe cognitive difficulties and/or cognitive communication difficulties.

Papers focussed exclusively on individuals with mild to moderate brain injuries were excluded. In cases where the severity of the acquired brain injury population was not detailed fully, was unclear or severity was mixed, papers were excluded if (1) less than 50% of the population was defined as having a severe brain injury, (2) the authors excluded participants who had impaired language, comprehension, or orientation, or the authors stated that participants were excluded if they were unable to complete mood and/or other measures, or (3) the reported cognitive assessment results or screening test results demonstrated the population had mild to moderately impaired cognition. All aspects of title searching, data extraction, risk of bias assessment, and analysis were performed by the principal researcher (AR). An additional researcher (SC) acted as a second reviewer for a randomly selected 10% of the screening.

### Evaluation of methodological quality

The COSMIN (Consensus-based Standards for the selection of health Measurement INstruments) risk of bias checklist (RoB) was used to examine the methodological qualities of the included studies based on the available information provided by the authors.^12^ A four-point rating scale is used to rate the quality of each study (“very good”, “adequate”, “doubtful”, or “inadequate”) using a “worst score counts” approach.^12^ Following completion of the risk of bias rating for the studies, each measure is rated using the criteria of good measurement properties as either sufficient (+ ), indeterminate (?) or insufficient ( − ).^13–15^ COSMIN suggests that outcome measures be chosen if there is evidence of (a) sufficient content validity and (b) at least low-quality evidence of sufficient internal consistency. They should not be recommended for use if they have high-quality evidence of an insufficient measurement property. Outcome measures can be tentatively recommended with suggestions for further research if they do not fall within the previously mentioned criteria (i.e., have mixed sufficient or indeterminate results).^13^

As there is no clear outcome measure that is considered a gold standard in this field, structured diagnostic interviews were accepted as the gold standard. In relation to construct validity (box 9), it was decided that correlations with other (established) instruments measuring mood should be ≥0.50, that correlations with instruments measuring unrelated constructs should be <0.30 and the area under curve (AUC) should be ≥0.70, as per suggestions made by the COSMIN manual.^12^

## Results

**Figure 1.**
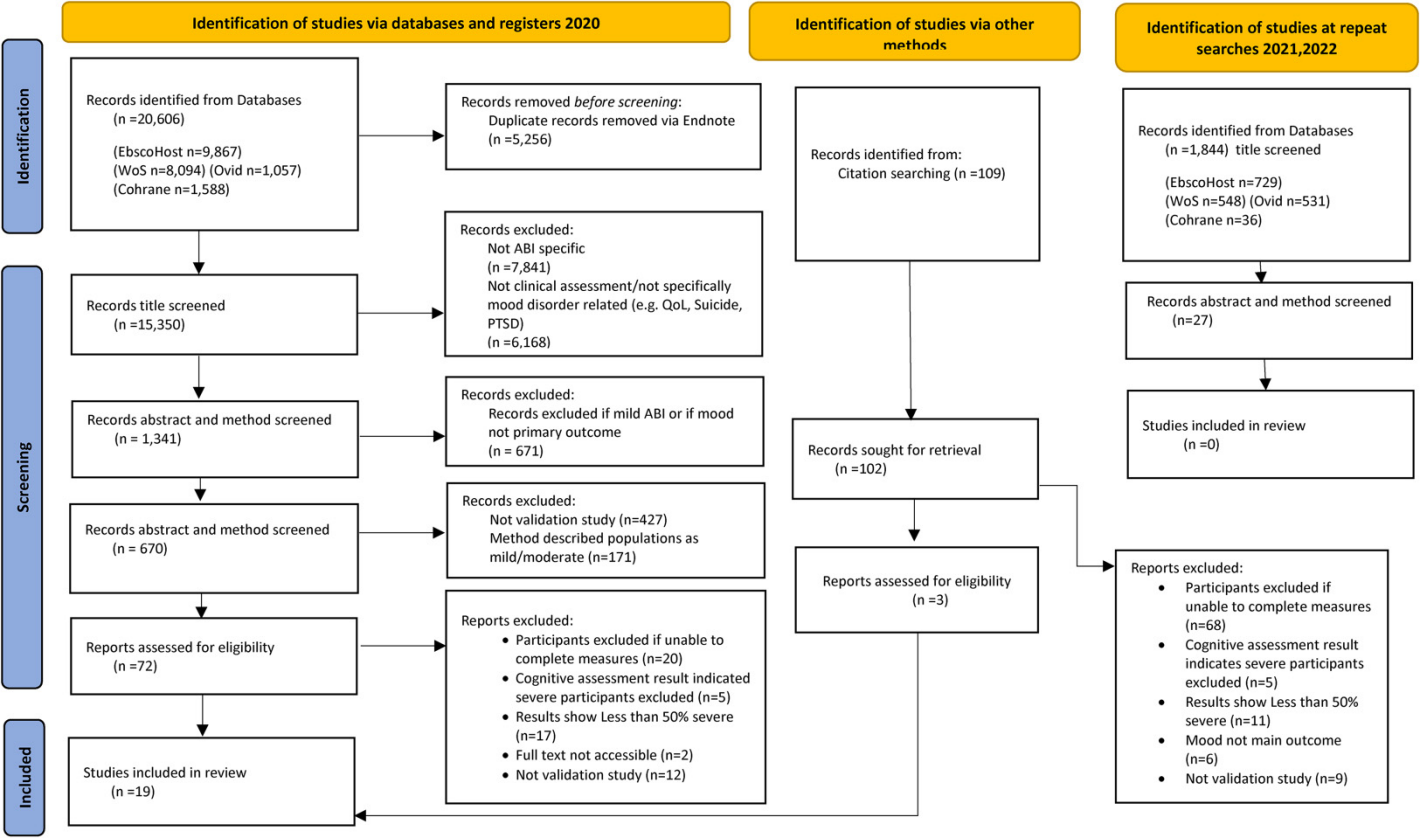
PRISMA flow chart showing numbers through article selection process.^8^ ABI = Acquired brain injury, PTSD = Post-traumatic stress disorder, QoL = Quality of life.

The initial searches yielded 20,606 articles. After duplicates were removed electronically via the Endnote database, 15,350 articles remained. Following systematic title and abstract screening, 72 articles were screened at full text level. Sixteen articles were retained for inclusion and, following a citation search, an additional three papers were included. Repeat searches in 2021 and 2022 did not yield any additional papers (See Figure 1). The final 19 articles, involving 2,914 participants, were included for analysis of risk of bias using the COSMIN risk of bias checklist (see Table 1).

**Table 1.** Description of severity, methodological quality, and COSMIN rating of included studies.^35^

Measure	Authors	sample (n severe ABI), setting, time after ABI	Measure of severity	Rate of depression	Gold standard	Methodological quality	Rating	
**ADRS**	Benaim et al.^16^	49 stroke (nm, 23 Aphasic), Rehabilitation centre, 66 + −39 days	Not specified by authors.	43% depressed according to psychologist.	Psychologist interview	Responsiveness – Doubtful. Day 0: r = 0.65, *p* < 10-6; Day 30: *r* = 0.64, *p* < 10-3).	Responsiveness	−
	France		Answers of 18% considered “doubtful” and sensitivity to change improved if these were excluded.					
	Laures-Gore et al.^17^	25 stroke (nm, all aphasic), SLT referrals, 2.70 years	Not specified by authors.	10/23 on ADRS, 12/24 on SADQ-10, 10/25 met threshold on both scales.	None, using diagnosis of depression as marker	Internal consistency - Very good Cronbach's alpha = 0.671, 0.742 with removal of item 9 Hypothesis testing – adequate positive correlation with SADQ-10 scores (*r* = 0.708, *p* < 0.001).	Internal consistency	+
							Hypothesis testing	+
			One patient had very severe aphasia, four had severe aphasia. Three unable to complete self-rating scale.					
	USA							
**BDI**	Schwarzbold et al.^18^	46 TBI patients, intensive care, (*n* = 46), 25 months (13–22.3)	GCS of <8	30.4% depressed	SCID interview DSM-IV	Criterion validity – very good. AUC = 0.946, p < 0.0001.	Criterion validity	+
			Assessed in chronic phase.					
	Brazil							
			13 patients could not be located or assessed.					
**BDI-II**	Siegert et al.^19^	315 inpatient, (nm), rehabilitation unit, 1 month-25 years (6 months)	Not specified by authors.	N/A	None	• Structural validity – Very good Chi Square = 86 (df72), *p* = 0.115. (*removal of items 18, 16, and 21- appetite, sleep, and interest in sex).^16^		
			Extreme scores excluded in Rasch analysis – unclear if related to ability to complete measures.				Structural validity	?

	UK								
	Siegert et al.^20^	353 inpatients (nm)	Not specified by authors.	N/A	None	Structural validity – Adequate Reliability coefficients for full BDI-II alpha = 0.89; cognitive/affective subscale (11 items) alpha = 0.85; and somatic subscale (eight items) alpha = 0.77 two factor solution (chi square difference = 0.551, *p* > 0.25) .^17^	Structural validity	+
	UK		Scores were calculated for 315 patients. Missing data on remainder – not clarified. Administered by psych/SLT and adapted.					
**CES-D**	Bush et al.^21^	253 TBI (*n* = 53%), patients referred for outpatient neuropsychologicaone evaluation, <1 month -> 2 years	GCS 3–8. Measures were self-report unless visual difficulties, a “few” were read to subjects by examiner.	58.1% were depressed on BDI, 48.4% on CES-D	None	Structural validity – Adequate 4 factor structure analysis accounts for 56.01% of variance. (1) Dysphoric affect, and somatic and retarded activity items. (2) Interpersonal difficulties and dysphoric affect. (3) All four positive affect items. (4) Three somatic and retarded activity items – decreased appetite, decreased talking, increased crying. Internal consistency – inadequate Coefficient alpha = 0.8195, Spearman brown split half reliability *r* = 0.8284. Hypothesis testing – very good concurrent construct validity with BDI (*r* = 0.673, *p* < 0.0001), MMPI-II D (*r* = 0.579, *p* = 0.15 and T scores (*r* = 0.536, *p* = 0.027).	Structural validity	−
							Internal consistency	+
	USA						Hypothesis testing	+
**CGI-S**	Laska et al.^22^	89 patients (nm), Stroke unit.	Not specified by authors.	12% cumulated frequency	DSM-IV	Criterion validity – Very good Cut-off ⩾2: Sensitivity: (Baseline = 0.33, 1 month = 3, months = 0.75, 6 months = 1); specificity: (Baseline = 0.97, 1 month = 0.93, 3 months = 0.94, 6 months = 0.92) PPV: (Baseline = 0.33, 1 month = 0.58, 3 months = 0.43, 6 months = 0.50).	Criterion validity	?
			26/80 (29%) lacked reliable yes/no capability; 76% could complete MADRS.					
	Sweden.							
**DASS-21**	Randall et al.^23^	Archival data of 504 rehabilitation patients, (nm), measures completed within 2 years of TBI	Length of PTA ranged from 24 h to 183 days (mean = 26.84, SD = 27.92), “…the current sample was generally representative of moderate to severe TBI”.	N/A	None	Structural validity – very good Quadripartite model best fit – depression, anxiety, stress, general distress (mean discrepancy = 779.75; CMIN/DF = 2.07; CFI = >0.95; RMSEA = 0.047; GFI = 0.94; SRMR = 0.029). Internal consistency – Very Good Good fit for all 4scales (>0.80). Factor structures inter-correlate (*p* < 0.001) strong relations between depression and anxiety subscales (*r* = 0.73), depression and stress subscales (*r* = 0.80) and anxiety and stress subscales (*r* = 0.73).	Structural validity	+
							Internal consistency	+
	Australia							
			PTA 1–4 weeks (47.82%), >4weeks (32.34%).					
**DISCS**	Turner-Stokes et al.^24^	114 (nm) ABI rehabilitation patients, median time since onset was 12 weeks	FIM + FAM assessments were available for 84 patients, showed moderate to severe difficulties, cognitive subscale score median 25 (IQR 20–32, 35 = unaffected); “in this cohort patients had relatively high skill and able to complete BDI-II, NGRS, and be categorised by DSM-IV interview”.	43 cases (39.8%)	BDI-II, DSM-IV	Reliability–doubtful Inter-rater, *N* = 66, quadratic weighted Cohen's k, DISCS *k* = 0.84, NGRS *k* = 0.84. Hypothesis testing – Adequate DISCS = *ρ* = 0.87, (NGRS), *ρ* = 0.66 (BDI-II), *ρ* = 0.59 (DSM-IV), NGRS *ρ* = 0.65 (BDI-II), *ρ* = 0.59 (DSM-IV). Responsiveness– doubtful N = 44, DISCS correlate with NGRS Spearman's *ρ* = 0.77, *p* < 0.001, and BDI-II (*ρ* = 0.38, *p* < 0.01).	Reliability	?
							Hypothesis testing	?
							Responsiveness	?
	UK							
**GHQ**	Kinsella et al.^25^	39 rehabilitation patients (*n* = 39),within 2 years of ABI	>24 h PTA, (29 >4 weeks PTA). 11 inpatients, 8 completed rehab, 20 outpatients.	59% considered "case” level for psychiatric disorder.	None	Reliability–doubtful Retest of *n* = 20, *r* = 0.67–0.92. Hypothesis testing–doubtful Correlated with observer completion of scale, *r* = 0.446, *p *= 0.007.^23^	Reliability	?
							Hypothesis testing	−
	Australia							
**GMS**	Lightbody et al.^26^	28 hospital inpatients (nm), 14–28 days post stroke.	Not specified by authors.	PCD = 7/28 (25%), GMS = 12/28(43%) MADR = 13/24 (54%)	Psychiatrist assessment according to ICD-10	Criterion validity - Doubtful Kappa 0.40, 95% CI 0.04–0.67. Sensitivity of 71% (CI 29–96%), Specificity of 67% (CI 43–85%), PPV of 2% (CI 15–72%), and NPV of **% (CI 62–98%). Overall efficiency (proportion correctly identified as positive or negative) 68% (CI 48–84%).	Criterion validity	−
			Report including severe cog/comm participants with relative/carer assent. Severity measured by RBMT – Proxy rating used for 7/28 (25%).					
	UK							
**HADS**	Dawkins et al.^27^	140 ABI patients at neurorehabilitation centre, (*n* = 76), mean time since injury 4.1 years (0.03–33.4 years)	Length of PTA or GCS (not fully detailed).	N/A	None	Structural validity – Adequate Using Eigenvalue>1; 3 factor structure accounts for 58.1% of variance (two items loading on third factor). Factor 1 = 5.29; factor 2 = 1.70; factor 3 = 1.14. ^22^	Structural validity	?
	UK							
	Schwarzbold et al.^18^	46 TBI patients, intensive care, (*n* = 46), 25 months (13–22.3)	GCS of <8	30.4% depressed	SCID interview DSM-IV	Criterion validity – very good AUC = 0*.*947, *p* < 0.0001.^15^	Criterion validity	+
			Assessed in chronic phase.					
	Brazil							
			13 patients could not be located or assessed.					
**HAM-D**	Schwarzbold et al.^18^	46 TBI patients, intensive care, (*n* = 46), 25 months (13–22.3)	GCS of <8	30.4% depressed	SCID interview DSM-IV	Criterion validity – very good AUC = 0.89, p < 0.0001.^15^	Criterion validity	+
			Assessed in chronic phase.					
			13 patients could not be located or assessed.					
	Brazil							
**LEEDS**	Kinsella et al.^25^	39 rehabilitation patients (*n* = 39),within 2 years of ABI	>24 h PTA, (29 >4 weeks PTA). 11 inpatients, 8 completed rehab, 20 outpatients.	59% considered "case” level for psychiatric disorder.	None	Reliability – doubtful Test re-test scores correlations in the range of, *r* = 0.67–0.92 (*n* = 20). ^23^ Hypothesis testing- doubtful Concurrent validity - Correlated with observer completion of scale, *r* = 0.658, *p* = 0.00.^23^	Reliability	?
							Hypothesis testing	?
	Australia							
**MADRS**	Lightbody et al.^26^	28 hospital inpatients (nm), 14–28 days post stroke.	Not specified by authors.	PCD = 7/28 (25%), GMS = 12/28 (43%) MADR = 13/24 (54%)	Psychiatrist assessment according to ICD-10	Criterion validity – very good Kappa 0.60, 95%, CI 0.04–0.67 Sensitivity of 100% (CI 59–100), Specificity of 65% (CI 38–86%), PPV of 54% (CI 25–81%), NPV 100% (CI 53–90%). Overall efficiency (proportion correctly identified as positive or negative) 75% (CI 53–90%). ^24^	Criterion validity	−
			Report including severe cog/comm participants with relative/carer assent. Severity measured by RBMT – proxy rating used for 7/28 (25%).					
	UK							
	Laska et al.^22^	89 patients (nm), Stroke unit.	Not specified by authors.	12% cumulated frequency	DSM-IV	Criterion validity - Very good Cut-off ⩾10: Sensitivity: (Baseline = 0.66, 1 month = 0.57, 3 months = 0.50, 6 months = 0.67); Specificity: (Baseline = 0.93, 1 month = 0.94, 3 months = 0.96, 6 months = 0.97). PPV (Baseline = 0.29, 1 month = 0.57, 3 months = 0.40, 6 months = 0.67). ^19^	Criterion validity	?
	Sweden.		26/80 (29%) lacked reliable yes/no capability; 76% could complete MADRS.					
**PHQ-9**	Cohen et al.^28^	381 TBI patients in rehabilitation hospitals (54.3% severe), variable time since injury	Not specified by authors.	23–26%	None	Hypothesis testing – Adequate When PHQ-9 somatic items removed- *r* = 0.92, *p* < 001. Agreement 82.9–84.7%. ^30^	Hypothesis testing	+
			“Confirmed with medical records”.					
	USA							
**SADQ-10**	Laures-Gore et al.^17^	25 stroke (nm, all aphasic), SLT referrals, 2.70 years	Not specified by authors.	10/23 on ADRS, 12/24 on SADQ-10, 10/25 met threshold on both scales	None, using diagnosis of depression as marker	Internal consistency - Very good Cronbach's alpha 0.793, 0.828 with removal of question 10.^14^ Hypothesis testing - Adequate SADQ-10: Positive correlation with ADRS scores, (*r* = 0.708, *p* < 0.001). Cronbach's 0.793, removal of Q10 = 0.828.^14^	Internal consistency	+
							Hypothesis testing	?
			One patient had very severe aphasia, four had severe aphasia. Three unable to complete self-rating scale.					
	USA							
**SADQ-21**	Sutcliffe et al.^29^	17 patients that could not complete standardised measures, (with carers) identified from Speech and language therapy registers, observer rated measure	Not specified by authors.	N/A	None	Reliability - Doubtful Spearman's Correlation *r* = 0.72, *p* < 0.001.^27^	Reliability	?
			Specifically looking for aphasic patients unable to complete measures, no detail of severity of injury or cognition.					
	UK							
**SADQ-H**	Bennett et al.^30^	100 stroke patients (nm), 2–4 weeks post stroke	Not specified by authors.	Of the 79 patients, 16 were depressed (20%) and 17 (22%) anxious.	None	Internal consistency – Very good (α = 0.84) ^26^ Hypothesis testing - Adequate Correlation with HADS-D *rs* = 0.52, *P* =<0.001 (significant at 1% level). ^26^	Internal consistency	+
							Hypothesis testing	−
			HADS completed by those with “no communication impairment”. 79/100 completed HADS (79%).					
	UK							
**SADQH-10**	Cobley et al.^31^	165 aphasia patients from stroke wards and community (nm). Majority in first 6 months of stroke.	Not specified by authors.	76/165 low mood (45%)	None	Structural validity – adequate three Eigenvalues accounted for 59.4% of variance (social interaction and physical pain, tearful, and loss of interest and motivation.)^25^ Internal consistency- doubtful Cronbach's alpha = 0.77, *r* = 0.75. ^25^ Hypothesis testing – doubtful correlation with HADS-D *r* = 0.53, *P* =<0.001 (significant at 1% level). ^26^	Structural validity	?
							Internal consistency	+
			Not clarified. Report getting assent from family for participants with severe aphasia.					
	UK						Hypothesis testing	−
	Bennett et al.^30^	100 stroke patients (nm), 2–4 weeks post stroke	Not specified by authors.	Of the 79 patients, 16 were depressed (20%) and 17 (22%) anxious.	None	Internal consistency – Very good Low (α = 0.68). ^26^ Hypothesis testing – Adequate Correlation with VAMS - sad *r* = 0.30, *p* < 0.001, Depression = *r* = 0.06, *p* = 0.59.^25^	Internal consistency	+
			HADS completed by those with “no communication impairment”. 79/100 completed HADS (79%).				Hypothesis testing	−
	UK							
**SIMS**	Gertler et al.^32^ AUS	61 TBI patients in the community (nm), 5.71 years post injury (mean)	PTA	20 were diagnosed with MDE via SCID (32.8%)	SCID	Reliability - Inadequate Repeated measures by time rs (p) SEM: SIMS verbal 61 (.00) 1.41; SIMS visual .70 (.00) 1.40. Z (p) Effect size: SIMS verbal − 3.21 (.00).42; SIMS visual − 3.38 (.00).44. ^31^ Measurement error - Inadequate Repeated measures by time rs (p) SEM: SIMS verbal 61 (.00) 1.41; SIMS visual .70 (.00) 1.40. Z (p) Effect size: SIMS verbal − 3.21 (.00).42; SIMS visual − 3.38 (.00).44. ^31^ Construct validity - Very good Correlation with MDE diagnostic status on the SCID-5 showed moderate point-biserial correlation coefficients with both SIMS-Verbal (*r* = − 0.51, *p* < 0.01) and SIMS Visual (*r* = − 0.55, *p* < 0.01) at Time 1. Visual. Time 1 (*n* = 61), Time 2 (*n* = 58). Hypothesis testing - Very good difference between MDE and no MDE. Mann- Whitney U. SIMS verbal − 3.84 (.00)/.49 (time 1); –3.89 (.00)/.50 (time 2). SIMS visual − 4.26 (.00)/.55 (time 1) –4.25 (.00)/.54 (time 2).^31^	Reliability	?
							Measurement error	?
							Criterion validity	−
			WHODAS divided into low (less impairment) and high group (more impairment), (high *n* = 29).					
							Hypothesis testing	+
**SDSS**	Lightbody et al.^33^	71 acute stroke patients in hospital (nm). Assessed by nurse and carer.	Not specified by authors.	SCID 35.2%, Nurse SDSS 46.5%, Carer SDSS 22/30 (73.3%)	SCID	Reliability - Doubtful Interrater ICC = 0.43, 95% (CI:0.09–0.68) - moderate at best ^28^ Criterion validity - Very good Nurses SDSS cut off>2 was: sensitivity >2 = 64% (CI:43–82%), Specificity = 61% (CI:45–75%), Efficiency (proportion correctly identified as positive or negative) = 62%(CI:50–73%). Carers cut off >2 Sensitivity = 90% (CI:55–100%), specificity = 35% (CI:15–59%), efficiency (proportion correctly identified as positive or negative) = 53% (CI:34–72%) ^28^	Reliability	−
							Criterion validity	−
			No exclusions, cognition measured by RBMT – 0–2 = severe, 3–6 = moderate impairment, 7–9 = poor memory, 10–12 = normal memory. Mean RBMT 12 (IQR 7–18)					
	UK							
	van Dijk et al.^34^	116 patients (nm), of which 53 (45.7%) with communicative impairment	Not specified by authors.	CIDI = 35.8% (*n* = 19) in patients with communicative impairment and 12.7% (*n* = 8) in patients who were able to communicate	Clinical interview	Internal consistency – Very good (*α* = 0.57) ^29^ Reliability - Doubtful inter-rater reliability of the SDSS (ICC = 0.80; 95% CI: 0.63–0.89) ^29^ Criterion validity - Very good The discriminatory power at a cut-off score of ⩾2 sensitivity of 0.74 (95% CI: 0.49–0.91), a specificity of 0.40 (95% CI: 0.23–0.58), a PPV of 0.41 (95% CI: 0.25–0.59), an NPV of 0.72 (95% CI: 0.47–0.90), and an area under the curve (AUC) of 0.58 (95% CI: 0.42–0.75). ^29^ Hypothesis testing - Adequate Correlation between the CIDI-relative and the SDSS (*rs* = 0.18, *P* = 0.30). correlation between the Barthel Index and the SDSS (*rs* = −0.33, *P* = 0.02) ^29^	Internal consistency	−
							Reliability	+
			Patients with communicative impairment had moderate to severe disability (median Barthel Index score of 5 (interquartile range (IQR) 9, range: 0–20))				Criterion validity	−
	The Netherlands						Hypothesis testing	−
	Bennett et al.^30^	100 stroke patients (nm)	Not specified by authors.	Of the 79 patients, 16 were depressed (20%) and 17 (22%) anxious.	None	Internal consistency – Very good Low (*α* = 0.53). ^26^ Hypothesis testing - adequate correlation between SDSS and HADS-D *rs* = 0.34, *P* = 0.004 (significant at 5% level). ^26^	Internal consistency	−
							Hypothesis testing	−
	UK		HADS completed by those with “no communication impairment”. 79/100 completed HADS (79%).					
**SDSS- Likert**	van Dijk et al.^29^	116 patients, of which 53 (45.7%) with communicative impairment	Not specified by authors.	CIDI = 35.8% (*n* = 19) in patients with communicative impairment and 12.7% (*n* = 8) in patients who were able to communicate.	Clinical interview	Internal consistency - Very good SDSS-Likert s *α* = 0.69. ^29^ Reliability - Doubtful Inter-rater reliability of SDSS-Likert (ICC = 0.66, 95% CI:0.46–0.80). ^29^ Criterion validity - Very good The discriminatory power at a cut-off score of ⩾2 sensitivity of 0.74 (95% CI: 0.49–0.91), a specificity of 0.36 (95% CI: 0.20–0.55), a PPV of 0.40 (95% CI: 0.31–0.49), an NPV of 0.71 (95% CI: 0.50–0.85), and an area under the curve (AUC) of 0.59 (95% CI: 0.42–0.75).^29^ Hypothesis testing - Adequate Correlation between the CIDI-relative and the SDSS-Likert (*rs* = 0.18, *P* = 0.30). correlation between the Barthel Index and the SDSS-Likert (*rs* = −0.30, *P* = 0.03). ^29^	Internal consistency	-
							Reliability	-
	The Netherlands.		Patients with communicative impairment (*n* = 53) had moderate to severe disability [median Barthel Index score of 5 (interquartile range (IQR) 9, range: 0–20)].					
							Criterion validity	-
							Hypothesis testing	-
			Population mean MMSE 24.1 [SD = 3.5, range = 8–30].					
**TBI-QoL-D**	Cohen et al.^30^	381 TBI patients in rehabilitation hospitals (54.3% severe), variable time since injury,	Not specified by authors.	23–26%	None	Hypothesis testing - Adequate Correlated with TBI-QoL *r* = 0.83, *p* < 001. ^30^	Hypothesis testing	+
			“Confirmed with medical records”.					
	USA							
**VAMS**	Bennett et al.^30^	100 stroke patients (nm), 2–4 weeks post stroke	Not specified by authors.	Of the 79 patients, 16 were depressed (20%) and 17 (22%) anxious.	None	Internal consistency - Very good Low (*α* = 0.71). ^26^ Criterion validity - Very good Correlation with HADS-D *rs* = 0.36, *P* < 0.001 (significant at 1% level). ^26^	Internal consistency	+
							Criterion validity	−
			HADS completed by those with “no communication impairment”. 79/100 completed HADS (79%).					
	UK							
	Benaim et al.^16^	49 stroke (nm, 23 Aphasic),	Not specified by authors.	43% depressed according to Psychologist.	Psychologist interview	Responsiveness - Doubtful D0: *r* = 0.71, *P* < 10-6, D30: *r* = 0.52, *P* > 10-3). ^13^	Responsiveness	−
		Rehabilitation centre, 66 + −39 days	Answers of 18% considered “doubtful” and sensitivity to change improved if these were excluded.					
	France							
**VAS-D**	Kinsella et al.^23^	39 rehabilitation patients (*n* = 39), within 2 years of ABI	>24 h PTA, (29 >4weeks PTA). 11 inpatients, 8 completed rehab, 20 outpatients.	59% considered "case “level for psychiatric disorder.	None	Reliability - Doubtful Re-test of *n* = 20, *r* = 0.67–0.92. ^23^ Hypothesis testing - Doubtful Correlated with observer completion of scale, *r* = 0.217, *p* = 0.217. ^23^	Reliability	?
							Hypothesis testing	−
	Australia							
**VASES**	Bennett et al.^26^	100 stroke patients (nm), 2–4 weeks post stroke	Not specified by authors.	Of the 79 patients, 16 were depressed (20%) and 17 (22%) anxious.	None	Internal consistency - very good low (*α* = 0.83). ^26^ Hypothesis testing - adequate correlation with HADS-D *rs* = − 0.52, <0.001 (significant at 1% level). ^26^	Internal consistency	+
							Hypothesis testing	−
	UK		HADS completed by those with “no communication impairment”. 79/100 completed HADS (79%).					

+ : sufficient, − : insufficient, ?: indeterminate.

ABI = Acquired brain injury, ADRS = Aphasia Depression rating scale, BDI = Beck Depression inventory, CES-D = Centre for epidemiologic studies depression scale, CGI-S = Clinical Global Impression rating scale for severity, CIDI-Composite international diagnostic interview, DASS-21 = Depression anxiety stress scales, DISCs = Depression Intensity Scale Circles, DSM-IV = Diagnostic and Statistical Manual for Mental Disorders IV, FIM + FAM = Functional independence measure + Functional Assessment Measure, GCS = Glasgow Coma Scale, GHQ = General Health Questionnaire, HADS = Hospital anxiety and depression scale, HAMD-D = Hamilton depression rating scale, ICD-10 = International classification of diseases, Leeds = Leeds scale for depression & anxiety, MADRS = Montgomery-Asberg Depression rating scale, Nm = not mentioned, PCD = psychiatrist clinical diagnosis, PHQ = Patient Hospital Questionnaire, PTA = post-traumatic amnesia, RBMT = Rivermead Behavioural memory test, SADQ = Stroke Aphasic Depression Questionnaire, SADQ-H = Stroke Aphasic Depression Questionnaire Hospital version, SCID = Structured clinical interview for DSM, SDSS = Signs of Depression scale, SIMS = Single Item Mood Scale, SLT = Speech and language therapist, TBI = Traumatic brain injury, TBI QoL-D = Traumatic Brain injury quality of life scale, VAMS = Visual analogue mood scale, VAS-D = Visual analogue scale for depression, VASES = Visual analogue of self-esteem scale.

The included papers provided details on 25 measures of mood and depression (descriptions of these measures are provided in Table 2). Of these measures, 15 are self-report (completed by the patient) and 10 are observer or clinician rated. One of the measures described (Leeds depression scale) is now obsolete and no longer used in practice. Of the included measures, 10 were specifically developed for a brain injury population. The remaining measures were developed from a psychiatric assessment based on the Diagnostic and Statistical Manual of Mental Disorders (DSM), or psychological models of depression, and were originally intended for use with the general or psychiatric patient population (Table 2). Eight papers used the accepted gold standard (structured diagnostic interview by a psychiatrist according to the Diagnostic and Statistical Manual of Mental Disorders criteria).

**Table 2.** Description of the mood assessment measures included in this review.

Measure	Abbreviation	Construct	Target population	Details	Availability	Administration time	Training	Rater	Cut Off
1. Aphasic depression rating scale	ADRS	Depression	Stroke	9 item external assessment	Freely available (internet)	NM	NM	Clinician	19/32
2. Beck depression inventory	BDI	Depression	General Population	21 items, self-report	Meant to purchase, is freely available	10 min	Not required*	Patient	0–10 absent or minimal depression; 10–18 mild to moderate depression; 19–29 is moderate depression; 30–63 is severe depression.
3. Beck depression inventory-II	BDI-II	Depression	General Population	21 items, self-report	Meant to purchase, is freely available	10 min	Not required*	Patient	0–13 none or minimal depression; 14–19 mild depression; 20–28 moderate depression; 29–63 severe depression.
4. Centre for epidemiologic studies depression scale	CES-D	Depression	General population	20 item self-rated, 4 point scale	Freely available (internet)	10–20minutes	None	Patient	>16 depression (higher scores = more symptomology)
5. Clinical global impression rating scale for severity	CGI-S	Mental illness	Psychiatric population	3 item observer rated; Clinician rated on 7 point scale	Freely available	Varies	NM	clinician	Between 1 and 2
6. Depression anxiety stress scales	DASS-21	Anxiety & Depression	General population	21 items self-rated	Freely Available	10–15 min	NM	Patient	10–13 mild, 14–20 moderate, 21–27 severe, 28< extremely severe
7. Depression intensity scale circles	DISCs	Depression	Acquired brain injury	Graphic scale with 6 circles with increasing proportion of dark shading, self-rated	Freely available	Less than 5minutes	NM	Patient supported by clinician	>/ = 2 indicates “caseness”
8. General health questionnaire	GHQ	Mental health	General population	30 Item self-report	Available for purchase	Approx. 5 min	NM	Patient	4 and above indicates "caseness"
9. Geriatric mental status schedule	GMSS	Geriatric mental health	Geriatric general population	Structured interview for psychopathology	Available for purchase	45 min	Required	Clinician interview self-report	Various interpretations related to specific diagnoses.
10. Hospital anxiety and depression scale	HADS	Anxiety & Depression	General medical population	14 scale item, 7 depression items, self-rated	Freely available (internet), recently need to purchase	2–5 min	Not required*	Patient	>11 indicates “caseness”
11. Hamilton depression rating scale	HAM-D	Depression	General Population	21 items, scored out of 17 items, clinician rated	Freely available	15–20 min	Not required*	Clinician	0–7 normal, 8–13 mild, 14–18 moderate, 19–22 severe, >23 very severe
12. Leeds scale for depression and anxiety	LEEDS	Depression	General population	15 item self-report	Unable to locate scale. Discontinued.	NM	NM	Patient	6 or 7 as cut off
13. Montgomery-Asberg depression rating scale	MADRS	Depression	General population	10 items, interviewer administered	Freely available	20–60 min	None required	Clinician	7–19 mild, 20–34 moderate, 35–60 severe depression
14. Patient hospital questionnaire	PHQ 9	Depression	General medical population	9 item self-rated	Freely available (internet)	NM	Not required*	Patient	1–4 Minimal depression
									5–9 Mild depression
									10–14 Moderate depression
									15–19 Moderately severe depression
									20–27 Severe depression
15. Stroke aphasic depression questionnaire	SADQ-21	Depression	Stroke patients with aphasia (community)	21 item observer rated	Freely available (internet)	4 min	Not required*	Clinician/observer	Scores range from 0–63 on the SADQ-21, with higher scores indicating higher levels of depression. No cut-off score has been established for this version of the SADQ.
16. Stroke aphasic depression questionnaire (10 item)	SADQ-10	Depression	Stroke patients with aphasia	10 item observer rated	Freely available (internet)	2 min	Not required*	Clinician/ Observer	Scores range from 0–30. A cut-off score of 14 out of 30 has been found to be optimal for detecting the presence of Depression
17. Stroke aphasic depression questionnaire hospital version	SADQH	Depression	Stroke patients with aphasia	21 item observer rated	Freely available (internet)	4 min	Not required*	Clinician/ Observer	Scores range from 0–63 on the SADQ-H, A cut-off score of 17/18 has been found optimal for detecting the presence of Depression.
18. Stroke aphasic depression questionnaire hospital version (10 item)	SADQH-10	Depression	Stroke patients with aphasia	10 item observer rated	Freely available (internet)	2 min	Not required*	Clinician/ Observer	Scores range from 0–30. A cut-off score of 5/6 has been found optimal for detecting the presence of Depression.
19. Single item mood scale	SIMS	Mood state	Traumatic brain injury patients	Emotion faces and numerical rating	Freely available (internet)	<5 min	Not required	Patient	Rates mood on scale of 1–10, measure change over time
20. Signs of depression scale	SDSS	Depression	Elderly medically ill patients	6 item observer rated	Freely available	<2 min	None required	Observer	>2 = probable depression
21. Signs of depression scale – Likert	SDSS- Likert	Depression	Elderly medically ill patients	6 item observer rated	Freely available	<2 min	None required	Observer	>2 = probable depression
22. Traumatic brain injury quality of life scale	TBI-QOL	Quality of life	Traumatic brain injury	separate scale	Not freely available	NM	NM	Patient	NM
23. Visual analogue mood scale	VAMS	Mood	Neurologically impaired adults	Visual self-assessment	Available for purchase	NM	Manual purchased	Patient	NM
24. Visual analogue scale for depression	VAS-D	Depression	General population	Visual self-assessment	Freely available	NM	Manual purchased	Patient	NM
25. Visual analogue of self-esteem scale	VASES	Mood and self esteem	Stroke patients	10-item visual analogue scale	Available for purchase	NM	Manual purchased	Patient	NM

NM = Not mentioned, *Instructions provided for administration on the measure.

The psychometric properties of the studies on all the instruments including risk of bias ratings for each study are detailed in Table 1. Due to the heterogeneity of the included acquired brain injury populations and the varied measures used as the gold standard, it was decided that meta-analysis was not appropriate and a narrative summary was completed. Additional data on population, location, and sample size was extracted. Of particular relevance to this review, data on the number of participants considered “severe” and how this severity was established was extracted.

### Severity

Severity of the acquired brain injury was adequately described in six papers (details provided on GCS or post-traumatic amnesia scores)^18,21,23,25,28,32^; one paper reported on disability outcome measures,^24^ the remaining 12 papers reported that they did not exclude severe acquired brain injury patients, but did not fully specify the severity of their included patient population meaning that the percentage that had severe cognitive impairments could not be established. These 12 papers were included as they did not report excluding patients who were severely impaired so could not be excluded from the review. However, the validity of the measures with severely impaired patients cannot be confidently determined from these papers as they had mixed severity participants.

### Structural validity

Structural validity, the degree to which scores on a scale reflect the unidimensionality of the construct being measured, was examined in studies of five measures (Table 1). Two papers, examining the Beck Depression Inventory-II (BDI-II) and the Depression and Anxiety Severity Scale-21 (DASS-21), calculated a statistic deemed appropriate by COSMIN ^19^^,^^23^ and met criteria for a ‘sufficient’ rating. Despite the Beck Depression Inventory-II study being rated as unbiased and having a sufficient rating, it must be noted that the authors suggested an 18-item measure for unidimensionality (removing three items that related to the somatic features of depression).^19^ The authors examining the Depression and Anxiety Severity Scale-21 recommended a four-factor structure (depression, anxiety, stress, and general distress) based on their examination of the records of 504 traumatic brain injury patients. Their method of examining records limited the ability to extract information on the cognitive impairments of those being assessed using this self-report measure. Four of the studies examining structural validity noted somatic symptoms or pain as a separate factor within the measure, which is likely to be an important consideration when assessing individuals post brain injury.

### Internal consistency, reliability, measurement error and responsiveness

Internal consistency, the degree that items are interrelated, was reported in 12 studies on ten measures. Nine of these studies were rated as ‘sufficient’ and had low risk of bias (Table 1). Six studies reported on test-retest reliability of measures, of which only one study examining the Signs of Depression Severity Scale was found to be ‘sufficient’, although this result was of low quality due to its risk of bias.^34^ One study reported measurement error (how close the scores of repeated measurements in stable patients are), ^32^ which was found to be inadequate due to scores not being stable. Due to the lack of reporting of the details required by COSMIN standards neither of the two papers reporting on responsiveness of the Aphasia Depression Rating Scale and Visual Analogue Mood Scale^16^ or Depression Intensity Scale Circles^24^ were found to be sufficient.

### Criterion validity and construct validity

Criterion validity is the degree to which the scores of a measure reflect those of the gold standard. Six papers reported on criterion validity, one of which was ‘sufficient’.^18^ Eight papers reported on construct validity. Of these, four studies were found to be ‘sufficient’ with a low risk of bias.^17,21,28,32^

### Risk of bias

A number of the studies were found to be at high risk of bias according to COSMIN criteria. This was due to factors such as small sample size, lack of detail on whether environments were controlled in test–retest, inappropriate statistical methods, inadequate time interval between repeat measures and bias in comparing subgroups (Table 1^16, 21,24, 25,26,29,32,33,34^). For many of these studies, a lack of detail about the statistical methods used and specific details in reporting of results required resulted in lower ratings. Later studies appeared to more frequently report their findings in line with COSMIN requirements, which may indicate improved quality of studies, or more likely, improved standards of reporting.

### Adaptations

It was noted that in a number of the included studies it was explicitly stated that the recommended method of administration of the measures was adapted to compensate for patients’ access and understanding difficulties such as visuospatial impairments, low education levels and slow processing speed.^18,21,24^ This included reading the measures to patients and prompting them for answers, taking additional time and using large print or printed responses to prompt participants. These adaptations arguably change the original instructions and therefore may impact the validity of these measures. Specifically, if they are adapted and administered at the same time as the clinical interview, there may be a high risk of bias.

### Quality of psychometric properties

Regarding the psychometric properties of the measures used in studies with populations with unspecified severity of cognitive and communication impairment, six papers showed high quality and sufficient results (Table 1). These studies examined the Aphasia Depression Rating Scale (very good internal consistency and hypothesis testing for construct validity, both rated as sufficient)^17^; the Depression Anxiety Severity Scale 21 item (very good structural validity and internal consistency, both rated as sufficient)^23^; the Stroke Aphasia Depression Questionnaire 10 item (two studies finding very good and sufficient internal consistency^17,30^ and adequate hypothesis testing for construct validity of sufficient or indeterminate quality); the Stroke Aphasia Depression Questionnaire Hospital version^30^ (with very good and sufficient internal consistency and hypothesis testing of construct validity); the Traumatic Brain Injury Quality of Life scale and the Patient Health Questionnaire nine item (both with very good and sufficient hypothesis testing of construct validity)^28^; the Beck Depression Inventory-II (with adequate and sufficient structural validity)^20^; the Visual Analogue Mood Scale and the Visual Analogue Self Esteem Scale^30^ (both with very good and sufficient internal consistency and adequate hypothesis testing for construct validity rated as indeterminate); and the Hamilton Depression Scale, Hospital Anxiety and Depression Scale and Beck Depression Inventory^18^ (all showing very good and sufficient criterion validity).

## Discussion

This review examined the psychometric properties of 25 mood measures reported in 19 studies of validity in people with severe or mixed severity acquired brain injury. A range of psychometric properties was reported and results ranged from “inadequate” to “very good”.

Due to the heterogeneity of the populations, variability in choice of gold standard, as well as the mixed results of studies on the same measures, it was not possible to pool the results. Research on stroke and research on traumatic brain injury are seen as separate fields due to the nature of prognosis and recovery patterns. This makes it challenging to find research on the impact of shared symptoms, i.e., cognitive impairments experienced due to neurological damage sustained due to the acquired brain injury.

The challenges of assessing mood after brain injury were seen in different ways in the selected papers. It was noted that the structural validity of the Depression Anxiety Severity Scale (DASS-21) and Stroke Aphasia Depression Questionnaire (SADQH-10-10) improved when somatic items were removed, possibly highlighting how these somatic symptoms overlap with physiological sequelae of acquired brain injury and may not specifically map on to mood-related issues. Papers examining observer or clinician-rated studies alluded to the issue of using measures with those with severe cognitive and communication impairments, stating that the need for the use of other-rated measures was due to issues with reliability when asking patients to complete self-report measures, usually in the context of communication impairment and aphasia.^17,30^ The authors of the Depression Intensity Scale Circles recognised that although the measure is designed to be used with the severely cognitively impaired, that the group with whom validity was examined had “relatively high skills and could complete (self-report measures)”.^24^ This further illustrates how the use of these measures require consideration in those with ongoing sequelae following their brain injury.

In completing this review it was noted that there were inconsistencies in how the term “severe” was used or operationalised by authors. Although authors defined the population as being “severe”, very few of them considered participants’ cognitive abilities and appeared to report on the severity of the acute injury as opposed to the associated impairments. In a study reporting on “severely injured” patients’ performance ^34^ the mean mini-mental status examination (MMSE) score was reported as 24/30 (25 and above is considered “normal”). This would indicate that the sample likely had moderate cognitive impairment rather than severe and that the studies were unlikely to have included the target population for this review. It was apparent that in many studies where the population was described as having a “severe” brain injury, the included patients had sufficient cognitive abilities to complete self-report measures via the reported administration (e.g., over the phone, patients returning measures independently via post, or patients providing informed consent). Papers tend to focus on injury severity and levels of consciousness at the time of the injury rather than the consequences of the acquired brain injury.

The lack of consistent reporting on the severity of the brain injury and the lack of consideration of those with ongoing severe cognitive and communication impairments highlights the paucity of research findings for this population. The authors of this paper who work clinically with people with severe cognitive and communication impairments following brain injury recognise that this population is not well defined in the literature and further studies need to better define this population. This lack of consistency in reporting also resulted in increased challenges in reviewing papers and reduced ability to synthesise results. Transparency in study inclusion and exclusion criteria is needed to improve the interpretability of data regarding patient populations in brain injury research. Researchers should consider including cognitive screens or assessments to determine the cognitive abilities of their study population, as the GCS and post-traumatic amnesia scores do not adequately measure persistent cognitive and communication impairments.

According to COSMIN guidance, the following measures appear to have robust evidence to support their use with people with mild to moderate brain injuries: the Aphasia Depression Rating Scale (ADRS), Depression Anxiety Severity Scale (DASS-21), Stroke Aphasia Depression Questionnaire (SADQH-10, SADQ-10, SADQH), Visual Analogue Mood Scale (VAMS) and Visual Analogue Self Esteem Scale (VASES). Although these measures have sufficient evidence for use with a less impaired acquired brain injury population, the available evidence does not show consistent or comprehensive results for use with those with severe cognitive and communication impairments following an acquired brain injury which puts limitations on their recommendation for this population. Additionally, there were issues regarding the adapted administration of measures to compensate for cognitive impairments, without consideration of how this impacts validity.

In terms of severe brain injury and the target population of this review, one study, which examined the Stroke Aphasia Depression Questionnaire (SADQ-10) and the Aphasia Depression Rating Scale (ADRS),^17^ showed evidence of sufficient psychometric properties of both measures *and* explicitly included those with severe cognitive impairments by examining patients with aphasia that limited their reliability in completing measures. While we tentatively recommend these two measures as appropriate clinician-rated measures for assessing mood in those with severe acquired brain injury, it should be noted that these results were with stroke patients only and that the sample size was very small (*n* = 25) and thus may not be generalisable. Due to the mixed results, heterogeneous populations, and lack of detail provided on severity, further validity studies on observer measures in a patients with evidence of severe cognitive/communication impairment are required. No self-report measures met criteria for recommendation.

A strength of this review is that a broad literature was searched, from database inception, which increased the likelihood that relevant papers were included. Additionally, the use of the COSMIN framework to extract relevant measurement properties and examine the risk of bias increased confidence in interpreting the results of the included studies.

A limitation is the majority of the searches and data extraction was completed by one researcher (AR), although reliability of study selection was established with a second reviewer (SC) at several points of the review. The cut-off of 50% of the population being considered to have suffered a severe acquired brain injury may have eliminated studies on small numbers of relevant patients, however, results from these studies would likely be less generalisable. The heterogeneity of the included studies limited synthesis of results.

## Conclusions

This review presents the psychometric properties of several measures of mood. However, very few can be recommended for use with people with ongoing severe cognitive and communication impairments following acquired brain injury. In particular, no self-report measures can be recommended for this population. The validity of one measure specifically designed for use with those with severe cognitive and communication impairments (depression intensity scales circles) has not been examined on the target population and further studies are recommended.

Clinician and observer rated measures are likely to be more appropriate in this population. The Stroke Aphasic Depression Questionnaire (10-item) and the Aphasia Depression Rating Scale showed sufficient internal consistency and construct validity in relation to assessment of patients with communication impairments and aphasias. Further studies of observer- and clinician-rated studies with larger sample sizes and other acquired brain injury populations are recommended.

Additionally, this review highlighted the issue with the definition of “severity” and that a severe brain injury and severe consequences of a brain injury are separate constructs and future research should provide clearer details when referring to “severity”. The standardisation of reporting with regards to the severity of the longer-term consequences of acquired brain injury for studies, and agreement of a gold standard for use in the brain injury population, would improve the interpretability of studies for future reviews.

Considering the current paucity of the evidence base, we recommend that patients with severe cognitive and communication impairments be assessed with caution using multiple sources of information (e.g., behavioural observation and collateral information) as well as clinician or observer rated measures such as the Stroke Aphasic Depression Questionnaire (10 item) or Aphasia Depression Rating Scale.

**Clinical messages**

- Mood and depression screens have not been sufficiently validated for use with those with severe cognitive and communication impairments following acquired brain injury.
- No self-report measures can be recommended for use with this population.
- Two observer rated mood scales (the Stroke Aphasic Depression Questionnaire 10 item and Aphasia Depression Rating Scale) can tentatively be recommended for screening mood issues in those with severe cognitive and communication impairments following acquired brain injury.

## Supplemental Material

sj-docx-1-cre-10.1177_02692155221139023 - Supplemental material for A systematic review of mood and depression measures in people with severe cognitive and communication impairments following acquired brain injury Click here for additional data file. Supplemental material, sj-docx-1-cre-10.1177_02692155221139023 for A systematic review of mood and depression measures in people with severe cognitive and communication impairments following acquired brain injury by Alexandra E. Rose, Breda Cullen, Sarah Crawford and Jonathan J. Evans in Clinical Rehabilitation
