# Supplementary material for: A systematic review of mood and depression measures in people with severe cognitive and communication impairments following acquired brain injury
Source: Clin Rehabil. 2022 Nov 15;37(5):679–700. doi: 10.1177/02692155221139023 (PMC10041571; doi:10.1177/02692155221139023)
Supplement: sj-docx-1-cre-10.1177_02692155221139023 - Supplemental material for A systematic review of mood and depression measures in people with severe cognitive and communication impairments following acquired brain injury [file sj-docx-1-cre-10.1177_02692155221139023.docx]

## **Appendix II: Search terms**

## **Cochrane Library: Cochrane Database for Systematic Reviews (CDSR) & Cochrane Central Register of Controlled Trials (CENTRAL)**

Last search run: 01.09.2022

S1 = “brain injury” or “acquired brain injury” or “traumatic brain injury” or “head injury” or “diffuse axonal injury” or “brain hypoxia” (TS) OR Stroke or “cerebrovascular accident” (TS)

S2= Mood or depress* or emotion* or affect* or “psychological distress” or “emotional distress” (TS) AND Assess* or screen* or measur* or evaluat* or rating or “assessment scale” (TS)

S3 = S1 AND S2

## **Web of science: Core collection & MEDLINE databases**

Last search run: 01.09.2022

S1 = “brain injury” or “acquired brain injury” or “traumatic brain injury” or “head injury” or “diffuse axonal injury” or “brain hypoxia” (TS) OR Stroke or “cerebrovascular accident” (TS)

S2= Mood or depress* or emotion* or affect* or “psychological distress” or “emotional distress” (TS) AND Assess* or screen* or measur* or evaluat* or rating or “assessment scale” (TS)

S3 = S1 AND S2

S4 = S3 NOT Animal or mouse or mice or rodent or veterinary

S5 = S4 NOT TS= (paediatric OR adolescent OR child OR infant)

S6 = S5 NOT TS= (Animal or veterinary or mouse or mice or rodent)

## **Ovid: Health and Psychosocial Instruments & Embase databases**

Last search run: 01.09.2022

S1 = TS = (Brain injury or head injury or traumatic brain injury or acquired brain injury or diffuse axonal head injury or brain hypoxia or TBI or ABI or DAI)

Indexes=SCI-EXPANDED, SSCI, A&HCI, CPCI-S, CPCI-SSH, BKCI-S, BKCI-SSH, ESCI, CCR-EXPANDED, IC Timespan=All years

S2 = TS= (Mood or emotion or feeling* or sad*or depress* or emotional distress)

Indexes=SCI-EXPANDED, SSCI, A&HCI, CPCI-S, CPCI-SSH, BKCI-S, BKCI-SSH, ESCI, CCR-EXPANDED, IC Timespan=All years

S3 = TS= (Assess* or measur* or evaluat*or scale or inventory or questionnaire or instrument or screen* or rating)

Indexes=SCI-EXPANDED, SSCI, A&HCI, CPCI-S, CPCI-SSH, BKCI-S, BKCI-SSH, ESCI, CCR-EXPANDED, IC Timespan=All years

S4 = AB= (Brain injury or head injury or traumatic brain injury or acquired brain injury or diffuse axonal head injury or brain hypoxia or TBI or ABI or DAI)

Indexes=SCI-EXPANDED, SSCI, A&HCI, CPCI-S, CPCI-SSH, BKCI-S, BKCI-SSH, ESCI, CCR-EXPANDED, IC Timespan=All years

S5 = AB= (Mood or emotion or feeling* or sad*or depress* or emotional distress)

Indexes=SCI-EXPANDED, SSCI, A&HCI, CPCI-S, CPCI-SSH, BKCI-S, BKCI-SSH, ESCI, CCR-EXPANDED, IC Timespan=All years

S6 = AB= (Assess* or measur* or evaluat*or scale or inventory or questionnaire or instrument or screen* or rating)

Indexes=SCI-EXPANDED, SSCI, A&HCI, CPCI-S, CPCI-SSH, BKCI-S, BKCI-SSH, ESCI, CCR-EXPANDED, IC Timespan=All years

S7 = #4 OR #1

Indexes=SCI-EXPANDED, SSCI, A&HCI, CPCI-S, CPCI-SSH, BKCI-S, BKCI-SSH, ESCI, CCR-EXPANDED, IC Timespan=All years

S8 = #5 OR #2

Indexes=SCI-EXPANDED, SSCI, A&HCI, CPCI-S, CPCI-SSH, BKCI-S, BKCI-SSH, ESCI, CCR-EXPANDED, IC Timespan=All years

S9 = #6 OR #3

Indexes=SCI-EXPANDED, SSCI, A&HCI, CPCI-S, CPCI-SSH, BKCI-S, BKCI-SSH, ESCI, CCR-EXPANDED, IC Timespan=All years

S10 = #9 AND #8 AND #7

Indexes=SCI-EXPANDED, SSCI, A&HCI, CPCI-S, CPCI-SSH, BKCI-S, BKCI-SSH, ESCI, CCR-EXPANDED, IC Timespan=All years

S11 = All= (Brain injury or head injury or traumatic brain injury or acquired brain injury or diffuse axonal head injury or brain hypoxia or TBI or ABI or DAI)

Indexes=SCI-EXPANDED, SSCI, A&HCI, CPCI-S, CPCI-SSH, BKCI-S, BKCI-SSH, ESCI, CCR-EXPANDED, IC Timespan=All years

S12 = TI= (Brain injury or head injury or traumatic brain injury or acquired brain injury or diffuse axonal head injury or brain hypoxia or TBI or ABI or DAI)

Indexes=SCI-EXPANDED, SSCI, A&HCI, CPCI-S, CPCI-SSH, BKCI-S, BKCI-SSH, ESCI, CCR-EXPANDED, IC Timespan=All years

S13 = #12 OR #11 OR #4 OR #1

Indexes=SCI-EXPANDED, SSCI, A&HCI, CPCI-S, CPCI-SSH, BKCI-S, BKCI-SSH, ESCI, CCR-EXPANDED, IC Timespan=All years

S14 = ALL= (Mood or emotion or feeling* or sad*or depress* or emotional distress)

Indexes=SCI-EXPANDED, SSCI, A&HCI, CPCI-S, CPCI-SSH, BKCI-S, BKCI-SSH, ESCI, CCR-EXPANDED, IC Timespan=All years

S15 = TI= (Mood or emotion or feeling* or sad*or depress* or emotional distress)

Indexes=SCI-EXPANDED, SSCI, A&HCI, CPCI-S, CPCI-SSH, BKCI-S, BKCI-SSH, ESCI, CCR-EXPANDED, IC Timespan=All years

S16 = #15 OR #14 OR #5 OR #2

Indexes=SCI-EXPANDED, SSCI, A&HCI, CPCI-S, CPCI-SSH, BKCI-S, BKCI-SSH, ESCI, CCR-EXPANDED, IC Timespan=All years

S17 = ALL= (Assess* or measur* or evaluat*or scale or inventory or questionnaire or instrument or screen* or rating)

Indexes=SCI-EXPANDED, SSCI, A&HCI, CPCI-S, CPCI-SSH, BKCI-S, BKCI-SSH, ESCI, CCR-EXPANDED, IC Timespan=All years

S18 = TI= (Assess* or measur* or evaluat*or scale or inventory or questionnaire or instrument or screen* or rating)

Indexes=SCI-EXPANDED, SSCI, A&HCI, CPCI-S, CPCI-SSH, BKCI-S, BKCI-SSH, ESCI, CCR-EXPANDED, IC Timespan=All years

S19 = #18 OR #17 OR #6 OR #3

Indexes=SCI-EXPANDED, SSCI, A&HCI, CPCI-S, CPCI-SSH, BKCI-S, BKCI-SSH, ESCI, CCR-EXPANDED, IC Timespan=All years

S20 = #19 AND #16 AND #13

Indexes=SCI-EXPANDED, SSCI, A&HCI, CPCI-S, CPCI-SSH, BKCI-S, BKCI-SSH, ESCI, CCR-EXPANDED, IC Timespan=All years

## **EBSCOHost: CINAHL, PsyArticles, PsycInfo & Psychology and behavioral sciences database**

- **CINAHL**

Last search run: 01.09.2022

S12 S3 AND S6 AND S9 Expanders - Apply equivalent subjects

Narrow by SubjectMajor: - stroke patients

Narrow by SubjectMajor: - brain injuries

Narrow by SubjectMajor: - depression

Narrow by SubjectMajor: - stroke

Narrow by SubjectAge: - all adult

S11 S3 AND S6 AND S9 Expanders - Apply equivalent subjects

Narrow by SubjectAge: - all adult

S10 S3 AND S6 AND S9 Expanders - Apply equivalent subjects

S9 S7 OR S8 Expanders - Apply equivalent subjects

S8 AB (Assess* or measur* or evaluat*) OR AB (scale or inventory or questionnaire or instrument or screen* or rating) Expanders - Apply equivalent subjects

S7 TX (Assess* or measur* or evaluat*) OR TX (scale or inventory or questionnaire or instrument or screen* or rating) Expanders - Apply equivalent subjects

S6 S4 OR S5 Expanders - Apply equivalent subjects

S5 AB (Mood or emotion or feeling* or sad*) OR AB (depress*) OR AB (emotional distress) Expanders - Apply equivalent subjects

S4 TX (Mood or emotion or feeling* or sad*) OR TX (depress*) OR TX (emotional distress) Expanders - Apply equivalent subjects

S3 S1 OR S2 Expanders - Apply equivalent subjects

S2 AB (Brain injury or head injury or traumatic brain injury or acquired brain injury or diffuse axonal head injury or brain hypoxia or TBI or ABI or DAI) OR AB (stroke or cerebrovascular accident or CVA) Expanders - Apply equivalent subjects

S1 TX (Brain injury or head injury or traumatic brain injury or acquired brain injury or diffuse axonal head injury or brain hypoxia or TBI or ABI or DAI) OR TX (stroke or cerebrovascular accident or CVA) Expanders - Apply equivalent subjects

- **APA PsycArticles, APA PsyInfo & Psychology and behavioural sciences database**

Last search run: 01.09.2022

Advanced Search Boolean/Phrase Interface

S23 S22 NOT SU (dementia or Alzheimer’s) - Boolean/Phrase Interface

S22 S21 NOT SU (animal or mouse or mice or rodent or veterinary)

S21 S20 NOT SU (paediatric or pediatric or child* or infant or adolescent)

S20 S9 AND S14 AND S19

S19 S15 OR S16 OR S17 OR S18

S18 SU Assess* or screen*

S17 TX Assess* or screen*

S16 SU Assessment Expanders - Apply equivalent subjects

S15 TX Assessment

S14 S10 OR S11 OR S12 OR S13

S13 SU Mood or depress* or emotion* or “psychological distress” or “emotional distress” Expanders - Apply equivalent subjects

S12 TX Mood or depress* or emotion* or “psychological distress” or “emotional distress” Expanders - Apply equivalent subjects

S11 SU mood

S10 TX mood

S9 S1 OR S2 OR S3 OR S4 OR S5 OR S6 OR S7 OR S8

S8 SU Stroke or “cerebrovascular accident”

S7 TX Stroke or “cerebrovascular accident”

S6 SU stroke

S5 TX stroke

S4 SU “brain injury” or “acquired brain injury” or “traumatic brain injury” or “head injury”

S3 TX “brain injury” or “acquired brain injury” or “traumatic brain injury” or “head injury”

S2 SU "brain injury"

S1 TX "brain injury" Expanders - Apply equivalent subjects
